# Clinical outcome of septic patients with undetectable vitamin D levels at ICU admission

**DOI:** 10.1186/2197-425X-3-S1-A80

**Published:** 2015-10-01

**Authors:** G De Pascale, MS Vallecoccia, E Gasperin, D Giacobelli, A Schiattarella, A Autunno, V Di Gravio, S Marsili, SL Cutuli, MA Pennisi, C Zuppi, SA Quraishi, M Antonelli

**Affiliations:** Department of Anesthesiology and Intensive Care, Sacro Cuore Catholic University, A. Gemelli Hospital, Rome, Italy; Institute of Biochemestry and Clinical Biochemestry, Sacro Cuore Catholic University, A. Gemelli Hospital, Rome, Italy; Department of Anesthesia, Critical Care and Pain Medicine, Harvard Medical School, Massachusetts General Hospital, Boston, United States

## Introduction

Septic patients with very low vitamin D (VD) levels are expected to most benefit from supplementation strategies but few data are available in this specific population.[1]

## Objectives

Our purpose is to investigate the clinical/epidemiological profile and sepsis-related outcome of critically ill septic patients with undetectable VD levels at ICU admission.

## Methods

We conducted an observational study enrolling, during a 12 months period, consecutive patients admitted to our ICU with severe sepsis/septic shock.

## Results

170 blood samples were obtained from 107 patients (septic shock / severe sepsis: 62% / 38%). ICU admission VD deficiency (≤ 20ng/mL) was observed in 93.5% of the patients: 57 (53.3%) showed undetectable levels ( < 7ng/mL). In patients (n = 33) who received, during the ICU stay, more than one VD blood sampling, hypovitaminosis D category did not change over time (p=ns). The principal infection site was the lung (48.6%): 50 (46.7%) patients were bacteraemic. Comparing patients with undetectable VD levels with those ones with values ≥ 7ng/mL, there were not significant differences regarding main comorbidities, presenting features and disease severity (*p*=ns). The former group showed a higher rate of microbiologically confirmed infections but a lower percentage of microbiological eradication (80.7% vs. 58%, *p* = 0.02; 35.3% vs 68%; *p* = 0.03, respectively). Furthermore they experienced longer duration of mechanical ventilation and vasopressor support: 9 *ds* [3.75-12.5] vs. 4 *ds* [2-0], *p* = 0.04; 7 *ds* [4-10] vs. 4 *ds* [2-7.25], *p*= 0.02. Sepsis-related mortality rate was higher in patients with VD levels < 7ng/mL (50.9% vs 26%). Multivariable regression analysis confirmed ICU admission undetectable VD concentration (p = 0.01) as independent predictor of sepsis-related mortality.

## Conclusions

Our results suggest that in critically ill septic patients undetectable VD levels at ICU admission may be a major determinant of clinical outcome. Further studies should assess the impact of replacement strategies in this subgroup of patients.
